# Supplementary material for: HIV-1 Rev interacts with HERV-K RcREs present in the human genome and promotes export of unspliced HERV-K proviral RNA
Source: Retrovirology. 2019 Dec 16;16:40. doi: 10.1186/s12977-019-0505-y (PMC6916052; doi:10.1186/s12977-019-0505-y)
Supplement: Supplementary file 1 — Additional file 1: Figure S1. Functional activity of HERV-K RcRE sequences with HIV-1 Revs. The functional activity of seven HERV-K RcREs was determined by transfecting the GagPol reporter constructs containing each identified RcREs as well as the control prRcRE into 293T/17 cells together with either 50 ng of prRev, prRec or one of three different HIV-1 Revs (1A, 6AG and 8G). After 72 h, cell supernatants were harvested and supernatant p24 was measured by ELISA. (A) Vectors expressing Rev from HIV 1A, 6AG and 8G, prRev or Rec were transfected together with reporter plasmids that contained the prRcRE or the RcREs from the seven indicated gene regions. Supernatant p24 was measured at 72 h post transfection. (B) For each of the Rev (or Rec) sequences, the activity of the Rev (or Rec)/prRcRE pair from part A was normalized to 1, and the activity of that specific Rev with each of the other RcREs was plotted relative to this value. This allows a direct comparison of the activity of each Rev (or Rec) protein with each of the different RcREs relative to its activity on the prRcRE. [file 12977_2019_505_MOESM1_ESM.pdf]

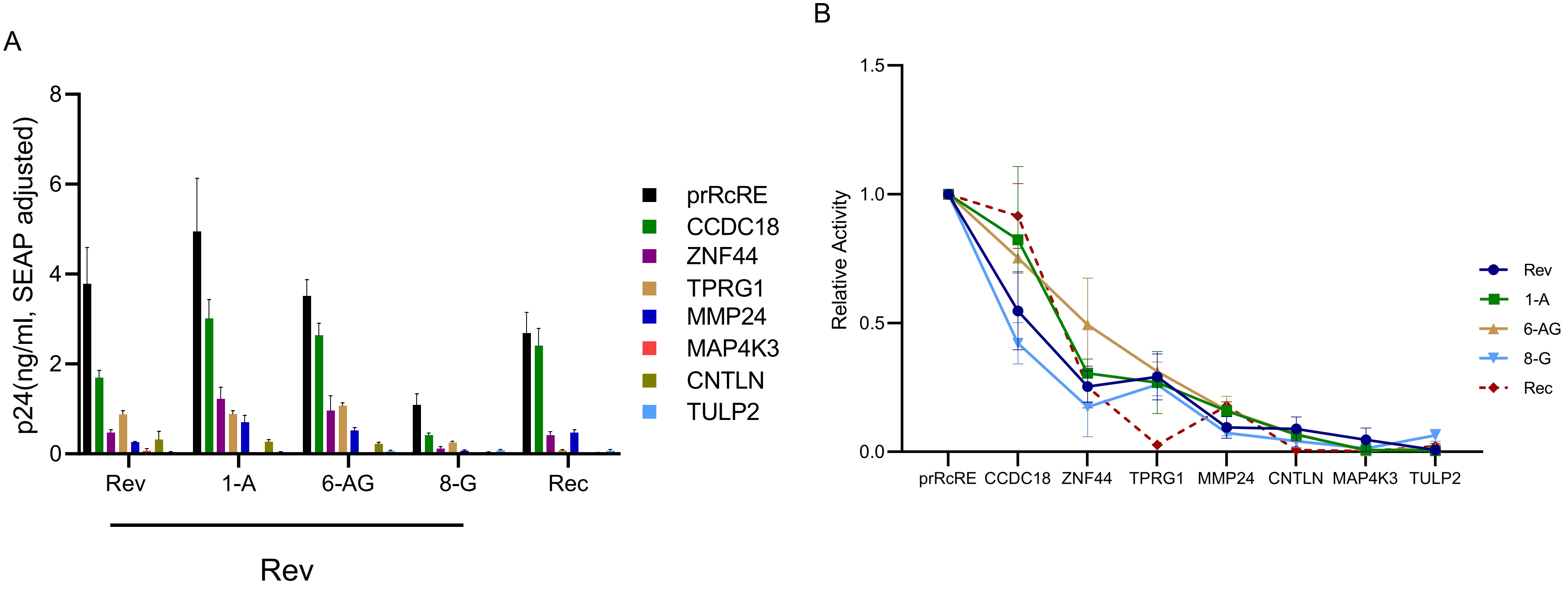

Figure S1. Functional activity of HERV-K RcRE sequences with HIV-1 Rev subtypes. The functional activity of seven HERV-K RcREs was determined by transfecting the GagPol reporter constructs containing each identified RcREs as well as the control prRcRE into 293T/17 cells together with either 50ng of prRev, prRec or one of three different HIV-1 Rev subtypes (1A, 6AG and 8G). After 72 hours, cell supernatants were harvested and supernatant p24 was measured by ELISA. (A) Vectors expressing Rev from HIV subtypes 1A, 6AG and 8G, prRev or Rec were transfected together with reporter plasmids that contained the prRcRE or the RcREs from the seven indicated gene regions. Supernatant p24 was measured at 72 hours post transfection. (B) For each of the Rev (or Rec) sequences, the activity of the Rev (or Rec)/ prRcRE pair from part A was normalized to 1, and the activity of that specific Rev with each of the other RcREs was plotted relative to this value. This allows a direct comparison of the activity of each Rev (or Rec) protein with each of the different RcREs relative to its activity on the prRcRE.
