# Supplementary material for: HIV-1 Rev interacts with HERV-K RcREs present in the human genome and promotes export of unspliced HERV-K proviral RNA
Source: Retrovirology. 2019 Dec 16;16:40. doi: 10.1186/s12977-019-0505-y (PMC6916052; doi:10.1186/s12977-019-0505-y)
Supplement: Supplementary file 2 — Additional file 2: Figure S2. Quantitative analysis of HERV-K proviral transcripts from total and cytoplasmic RNAseq data. After normalization of the data, the fold difference in the number of unique reads mapping to the 22q11.23 (left panel) or 4p16.1b (right panel) loci were quantified using DESeq2 for total or cytoplasmic RNA from the Rev, Tat, Tat and Rev or Rec transduced samples, compared to the samples transduced with the empty vector. [file 12977_2019_505_MOESM2_ESM.pdf]

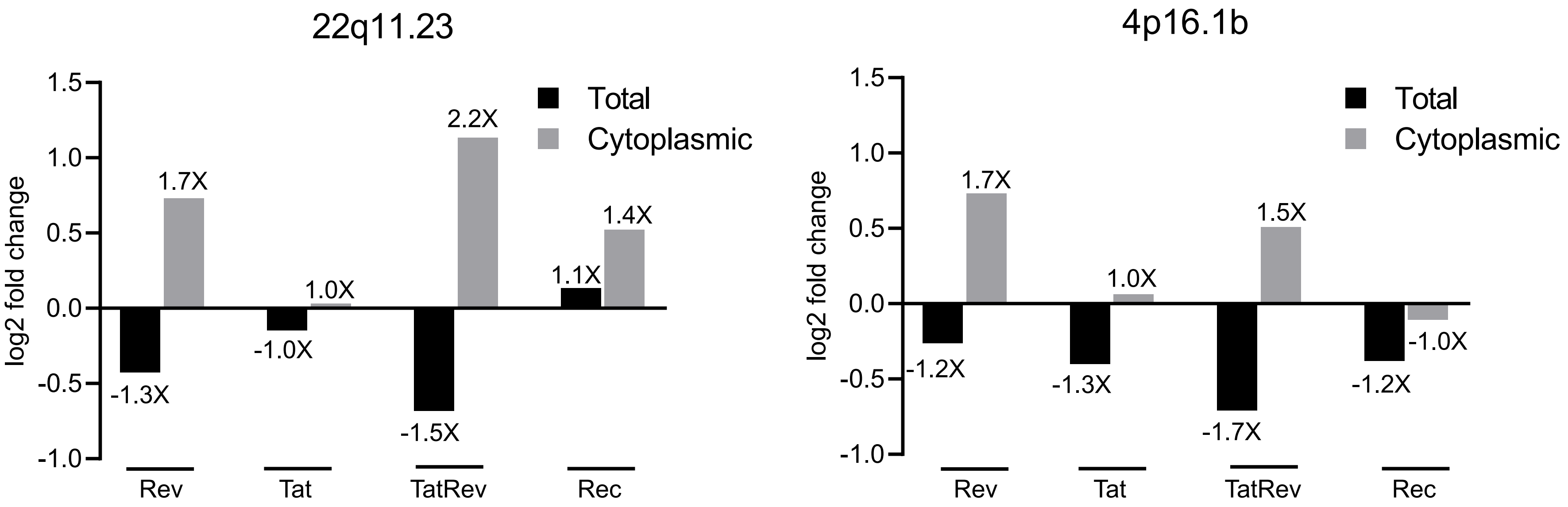

Figure S2. Quantitative analysis of HERV-K proviral transcripts from total and cytoplasmic RNAseq data. After normalization of the data, the fold difference in the number of unique reads mapping to the 22q11.23 (left panel) or 4p16.1b (right panel) loci were quantified using DESeq2 for total or cytoplasmic RNA from the Rev, Tat, Tat and Rev or Rec transduced samples, compared to the samples transduced with the empty vector.
