# Supplementary material for: HIV-1 Rev interacts with HERV-K RcREs present in the human genome and promotes export of unspliced HERV-K proviral RNA
Source: Retrovirology. 2019 Dec 16;16:40. doi: 10.1186/s12977-019-0505-y (PMC6916052; doi:10.1186/s12977-019-0505-y)
Supplement: Supplementary file 3 — Additional file 3: Figure S3. Visualization of NEAT1 reads from total and cytoplasmic RNAseq data. Total and cytoplasmic DESeq2 normalized read counts for NEAT1 were visualized with IGV. Note that DNA reads from total RNA map across the entire NEAT1 gene region and include both the NEAT 1_1 and NEAT 1_2 RNA isoforms. In contrast, most of the reads from cytoplasmic RNA map only to the region corresponding to the NEAT1_1 RNA isoform. [file 12977_2019_505_MOESM3_ESM.pdf]

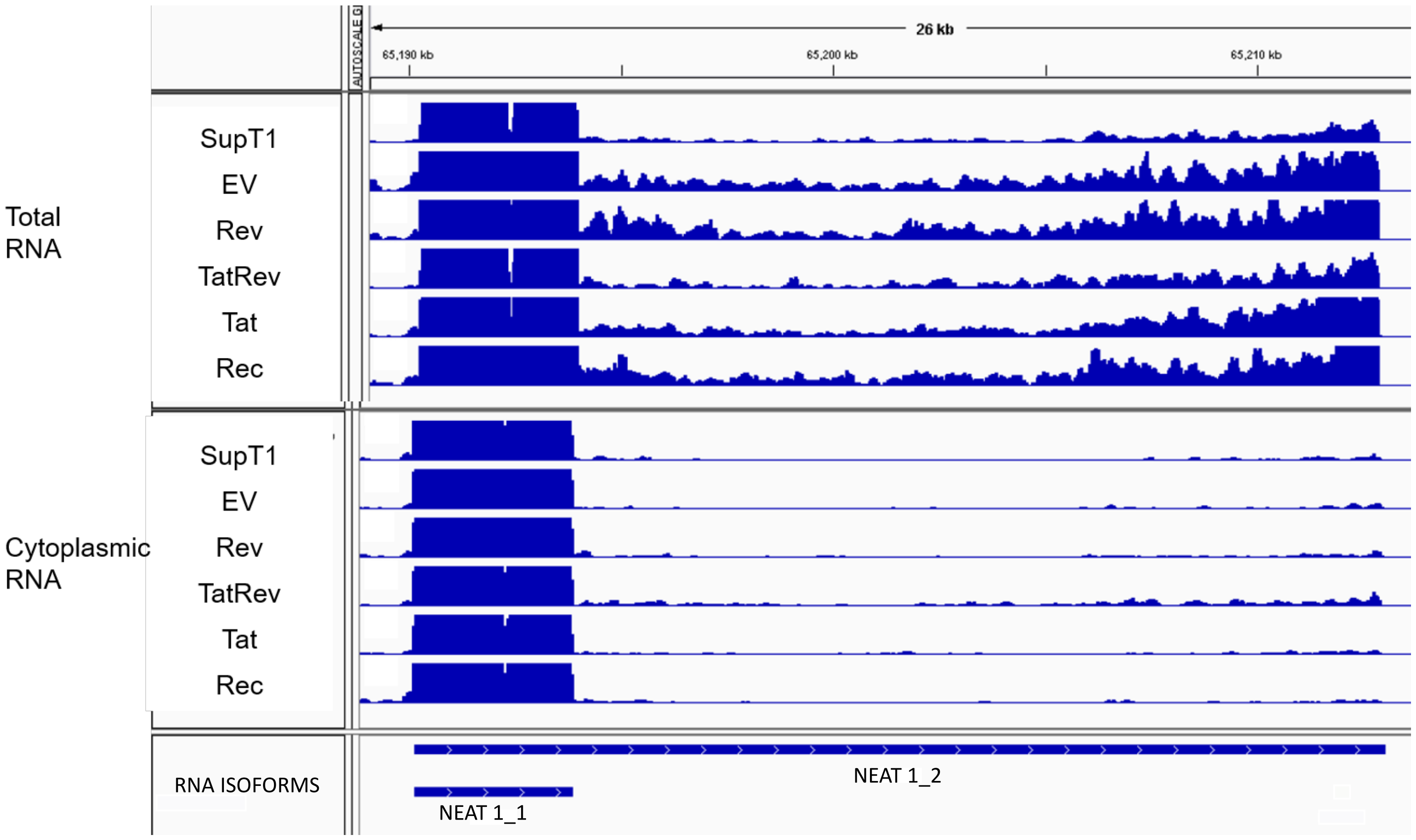

Figure S3. Visualization of NEAT1 reads from total and cytoplasmic RNAseq data. Total and cytoplasmic DESeq2 normalized read counts for NEAT1 were visualized with IGV. Note that DNA reads from total RNA map across the entire NEAT1 gene region and include both the NEAT 1_1 and NEAT 1_2 RNA isoforms. In contrast, most of the reads from cytoplasmic RNA map only to the region corresponding to the NEAT1_1 RNA isoform.
